# Greater variability in daily sleep efficiency predicts depression and anxiety in young adults: Estimation of depression severity using the two-week sleep quality records of wearable devices

**DOI:** 10.3389/fpsyt.2022.1041747

**Published:** 2022-11-07

**Authors:** Jae-A Lim, Je-Yeon Yun, Soo-Hee Choi, Susan Park, Hye Won Suk, Joon Hwan Jang

**Affiliations:** ^1^Department of Psychiatry, Seoul National University Health Service Center, Seoul, South Korea; ^2^Department of Psychology, Sogang University, Seoul, South Korea; ^3^Institute for Hope Research, Sogang University, Seoul, South Korea; ^4^Seoul National University Hospital, Seoul, South Korea; ^5^Yeongeon Student Support Center, Seoul National University College of Medicine, Seoul, South Korea; ^6^Department of Psychiatry, Seoul National University College of Medicine, Seoul, South Korea; ^7^Department of Human Systems Medicine, Seoul National University College of Medicine, Seoul, South Korea

**Keywords:** sleep efficiency, variability, depression, wearable device, dynamic structural equation models

## Abstract

**Objectives:**

Sleep disturbances are associated with both the onset and progression of depressive disorders. It is important to capture day-to-day variability in sleep patterns; irregular sleep is associated with depressive symptoms. We used sleep efficiency, measured with wearable devices, as an objective indicator of daily sleep variability.

**Materials and methods:**

The total sample consists of 100 undergraduate and graduate students, 60% of whom were female. All were divided into three groups (with major depressive disorder, mild depressive symptoms, and controls). Self-report questionnaires were completed at the beginning of the experiment, and sleep efficiency data were collected daily for 2 weeks using wearable devices. We explored whether the mean value of sleep efficiency, and its variability, predicted the severity of depression using dynamic structural equation modeling.

**Results:**

More marked daily variability in sleep efficiency significantly predicted levels of depression and anxiety, as did the average person-level covariates (longer time in bed, poorer quality of life, lower extraversion, and higher neuroticism).

**Conclusion:**

Large swings in day-to-day sleep efficiency and certain clinical characteristics might be associated with depression severity in young adults.

## Introduction

Young adulthood features psychological and environmental changes sometimes associated with the onset of depression and/or insomnia (1). A longitudinal epidemiological study of young adults found that those with lifetime histories of insomnia and/or hypersomnia evidenced higher rates of major depression and other psychiatric disorders (2). Insomnia or hypersomnia almost every day is one of the features of depressive symptoms (3), and existing literature on the link between depression and sleep disturbance indicates that depression and sleep disturbance are bidirectionally related (4–6). Sleep disturbance may be prodromal in terms of depression development (6, 7); sleep problems often precede depressive episodes (8). Sleep disturbances must be carefully evaluated when diagnosing depression.

Night-to-night sleep variability is an important second dimension of the sleep domain; individual-level sleep mean is the first dimension (9–11). Although sleep may vary from day-to-day, most research has focused on an individual average sleep (9). However, sleep variability is more predictive of various medical conditions than is the average sleep level (9, 12). Greater daily sleep variability may be associated with adverse health outcomes such as maladaptive health behaviors and impaired physiological processes (13). It may be considered to quantify the variability in daily sleep quality as a method of measuring sleep disturbance when diagnosing depressive symptoms.

Sleep quality is an important predictor of depression onset or recurrence (14, 15). Sleep quality is measured by assessing daily sleep variability either subjectively or objectively (16). Subjective tests include self-report questionnaires such as the Pittsburgh Sleep Quality Index (PSQI) (17). The global PSQI score is a well-established index of subjective sleep quality that reliably estimates the risk for depressive symptoms in general adult populations (18). The test is widely used because it is inexpensive and easy to administer, but it does not yield objective sleep data. Such data can be obtained via polysomnography (PSG), this is a typical test that can systematically examine objective sleep quality, but PSG requires a lot of resources for testing (19, 20). There is a need for alternatives that measure sleep quality in a cost-effective manner.

Wearable devices (sometimes termed fitness trackers) monitor health signals and track behaviors such as sleep patterns when assessing long-term disease progression or treatment responses (21, 22). Such devices yield data on individual sleep macrostructures (20), but sensor accuracy and reliability, and the algorithm used, must be considered (21). Such devices do not estimate objective sleep quality as accurately as PSG (20, 22, 23). The devices are worn on the wrist and measure natural sleep patterns; the data quality is inevitably inferior to that of PSG performed in a sophisticated setting. However, the devices enable longitudinal tracking of individual sleep patterns, thus capturing day-to-day sleep variability that PSG does not measure.

To the best of our knowledge, few studies have used wearable devices to examine the association between the severity of depression/anxiety, and daily sleep efficiency, particularly in terms of intra-individual variability of sleep efficiency over a relatively short period. This is our topic here. Although young adulthood is the peak period of new-onset major depression (2), young adults rarely seek treatment because most are unaware of their symptoms (24). Young adults are thus the optimal population for an intensive longitudinal study of the association between daily sleep variability and depression.

## Materials and methods

### Participants

Data were collected from June 2018 to October 2020 from 104 undergraduate and graduate students (42 males and 62 females). All completed mental health screening during regular on-campus checkups. We emailed those at risk for depression (to ask them to volunteer for the study); the control (CON) group was enrolled via leaflet advertising. The inclusion criteria were age 18–35 years, no prior diagnosis of a psychotic or substance use disorder, no prior loss of consciousness caused by a severe head injury, and no psychotropic drug use within 8 weeks prior to enrollment. Diagnoses of major depressive disorder (MDD) or mild depressive symptoms (MDS) were made by licensed psychiatrists based on semi-structured MINI-International Neuropsychiatric Interviews. The classification criteria of the MDD and MDS participants have been reported as in previously published manuscripts (24, 25). The MDD and MDS participants met at least one of the following criteria: Patient Health Questionnaire-9 ≥ 10 points; General Anxiety Disease-7 ≥ 10 points; State-Trait Anxiety Inventory-State ≥ 61 points (for males) or ≥65 points (for females); a history of at least 1 suicidal thought/attempt/plan within the past 6 months. Furthermore, the MDD/MDS participants experienced one or both of the following symptoms: (1) a depressive mood or (2) loss of interest or pleasure in daily life over the last 2 weeks. The CON group lacked any prior or current psychiatric disorder. All participants provided written informed consent prior to participation. This study was approved by the Institutional Review Board of the Seoul National University College of Medicine and Hospital (Seoul, Republic of Korea; approval no. 1608–079–785) and conducted to the ethical standards of the 1964 Declaration of Helsinki and its later modifications.

### Measures

Objective sleep-related variables were acquired daily through wearable devices, and demographic and clinical information were gathered using one-off self-report questionnaires. Thus, the objective sleep variables exhibit day-level data structures, and the demographic and clinical scores have person-level data structures.

### Objective sleep-related indices

Objective sleep data were collected using wearable devices, Fitbit Charge 2 (Fitbit Inc.); these are wearable wristbands that track daily activity levels and sleep patterns (20). All participants installed the Fitbit app on their mobile phones and synced the app to Fitbits provided by the researchers. All participants were instructed to wear the Fitbits for 2 weeks and then return them. We extracted sleep efficiencies and times in bed.

#### Sleep efficiency (%)

This measure of sleep quality is calculated using a combination of participant movements and heart-rate patterns and is not the same as the sleep score of the mobile application (16, 26). Scores range from 0 to 100%; a score of at least 85% reflects good sleep quality (27).

#### Time in bed (min)

This included bed times when both asleep and awake (16, 26).

### Clinical assessment

#### Patient Health Questionnaire-9

The Patient Health Questionnaire-9 (PHQ) (28, 29) screens for depression and its severity. A score of at least 10 generally indicates severe depression. Cronbach’s α for the 9 items was 0.85.

#### Generalized Anxiety Disorder-7

The Generalized Anxiety Disorder-7 (GAD) (30, 31) is a self-report instrument assessing anxiety; higher scores indicate greater anxiety. Cronbach’s α for the 7 items was 0.90.

#### World Health Organization quality of life abbreviated version

The World Health Organization Quality of Life Abbreviated Version (QOL) (32, 33) measures social relationships and physical and psychological health in the context of a cultural environment. A higher score indicates a better quality of life. The QOL features 26 items (α = 0.91); we used the total QOL score.

#### Barratt Impulsiveness Scale

The Barratt Impulsiveness Scale (BIS) (34, 35) assesses attentional, motor, and non-planning impulsiveness; greater scores indicate more impulsive behaviors and preferences. We used the BIS total 30-item score (α = 0.86).

#### NEO Five Factor Inventory

The NEO Five Factor Inventory (NEO) (36, 37) examines the Big Five personality traits (agreeableness, conscientiousness, extraversion, neuroticism, and openness to experience); we used the 60-item (shorter) inventory. There were 12 items in each of the agreeableness subscale (α = 0.68), the conscientiousness subscale (α = 0.81), the extraversion subscale (α = 0.81), the neuroticism subscale (α = 0.86), and the openness to experience subscale (α = 0.72).

#### Pittsburgh sleep quality index

The PSQI (17, 38) evaluates sleep quality over the past month; a global score of at least six indicates poor sleep quality (7 items; α = 0.61). We used the global PSQI score.

### Covariates

We adjusted for sex, age, time in bed, the QOL, the NEO personality traits, and the BIS and PSQI scores; all are associated with depression or anxiety (39–44). Note that the “Time in Bed” index (“BED” in Table 1) served as a person-level variable during analysis, although it was measured daily. We calculated the individual mean scores because all other covariates and outcome variables were person-level variables.

**TABLE 1:** Descriptive statistics, ANOVA, and chi-squared test results.

Day-level variable									
	**Overall (*n* = 2129)**		**MDD (*n* = 1023)**		**MDS (*n* = 661)**		**CON (*n* = 445)**		***P*-value**
	***Mean***	***SE***	***Mean***	***SE***	***Mean***	***SE***	***Mean***	***SE***	

Sleep efficiency (%)	94.30	0.19	94.18	0.28	94.41	0.35	94.44	0.42	0.820

***Person-level variable***									

	**Overall (*N* = 100)**		**MDD (*N* = 48)**		**MDS (*N* = 31)**		**CON (*N* = 21)**		***P*-value**
**Sex**									
Male	40 (40.00%)		22 (45.83%)		10 (32.26%)		8 (38.10%)		0.476
Female	60 (60.00%)		26 (54.17%)		21 (67.74%)		13 (61.90%)		

	***Mean***	***SD***	***Mean***	***SD***	***Mean***	***SD***	***Mean***	***SD***	

Age	24.33	3.18	24.10	2.83	24.58	3.33	24.48	3.79	0.791
BED (min)	379.98	52.32	380.85	51.45	378.90	42.99	379.60	67.48	0.987
PHQ	8.77	5.12	11.06	4.61	8.81	4.48	3.48	2.77	<0.001
GAD	6.04	5.04	7.90	4.90	6.26	4.89	1.48	1.72	<0.001
QOL	48.39	9.61	44.06	7.82	49.13	9.62	57.19	6.94	<0.001
NEO1	39.05	5.96	38.85	6.30	39.45	5.97	38.90	5.35	0.904
NEO2	37.14	7.17	35.48	6.64	37.81	8.28	39.95	5.68	0.046
NEO3	34.07	6.96	33.15	7.34	33.00	6.62	37.76	5.42	0.022
NEO4	41.94	8.33	45.25	7.48	41.42	6.82	35.14	8.19	<0.001
NEO5	41.62	6.64	41.81	7.05	41.52	6.16	41.33	6.67	0.958
BIS	66.99	11.68	67.48	10.96	67.81	11.90	64.67	13.14	0.591
PSQI	7.83	2.85	8.44	2.71	7.23	3.06	7.33	2.65	0.121

The *P*-value reported in the sleep efficiency variable is a random ANOVA result that take into account the nested structure of the data, and the *P*-value described in the Sex variable is a result of the chi-squared test; MDD, major depressive disorder; MDS, mild depressive symptoms; CON, control; BED, time in bed; PHQ, Patient Health Questionnaire-9; GAD, General Anxiety Disease-7; QOL, WHO quality of life; NEO1, NEO agreeableness; NEO2, NEO conscientiousness; NEO3, NEO extraversion; NEO4, NEO neuroticism; NEO5, NEO openness to experience; BIS, Barratt Impulsiveness Scale-11; PSQI, Pittsburgh Sleep Quality Index.

### Data analyses

Before analysis, we extracted day-level sleep data from json files and created the “day” variable as follows. It was a sequential number according to the “date of sleep” and “start time (of sleep)” log recorded for each sleep event per participant. Then, we merged the sleep data with the person-level demographic and clinical data. Note that the “day” variable does not have an equal time interval for each individual as well as across different individuals. However, unequal time intervals were not an issue for our analysis since we did not examine lagged effects such as the association between yesterday’s sleep efficiency on today’s. Also, we obtained descriptive statistics for all participants and the three groups. To examine the construct validity of the measures, random and one-way ANOVA, the chi-squared test, and correlation analysis were performed.

It is of our main interest that the association between intra-individual variability in sleep efficiency and depression/anxiety is investigated. For this purpose, a variability measure should be obtained for sleep efficiency. The majority of research on intra-individual variability in sleep patterns have calculated intra-individual standard deviation (iSD) as a variability measure, which is simply the standard deviation of the observed data for each person. Although simple and intuitively appealing, using iSD is not optimal for unbalanced data as ours, in which individuals have differing numbers of observations. The obtained iSD is less reliable for individuals with a smaller number of observations (45). To address this issue, we used dynamic structural equation modeling (DSEM) (46). DSEM can treat individual mean and individual variance (specifically, log-transformed individual variance) of daily measures as latent variables. This allows researchers to include these latent variables as a predictor or an outcome in a model with other variables, and thus to examine the relationship between the variables more reliably. In addition, unlike the two-step iSD approach (the iSD is first calculated and then the relationship between iSD and other variables is examined), DSEM proceeds in a single step.

Using DSEM we constructed a model in which the PHQ and GAD were outcomes, and each outcome was regressed on individual mean and (log-transformed) individual variance of daily sleep efficiency. The individual mean sleep efficiency was included as a predictor as well as the individual variance considering that individual mean and individual variance tend to be correlated although not completely overlapping (9). To control for the effects of covariates including sex, age, time in bed, the QOL, NEO personality traits, BIS, and PSQI, these variables were also included as predictors in the model.

Sleep data extraction, descriptive statistics, random and one-way ANOVA, chi-squared test, correlation analyses, and graphical representations were performed using R version 4.1.2 (47), and DSEM analysis employed M*plus* version 8.7 (48). In ANOVA, chi-squared test, and correlation analyses, *P*-values < 0.05 were considered to indicate statistical significance. In DSEM, Bayesian estimation with MCMC was used and a posterior distribution was provided for each parameter. Therefore, we considered it statistically significant if the 95% credible interval for a parameter does not contain 0.

## Results

Data from 100 participants were analyzed; we excluded data from two of the MDS and two of the MDD groups who considered issues with sensor wearing (recorded sleep efficiency less than 3 days) or were outliers (deviating three standard deviations from the mean sleep efficiency of all participants). The descriptive statistics, the random and one-way ANOVA, and chi-square test results are presented in Table 1.

**FIGURE 1:**
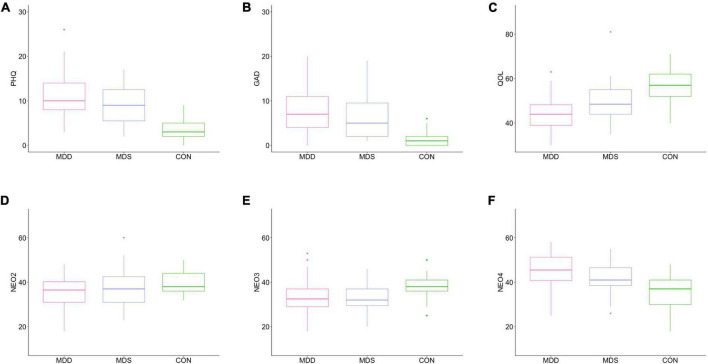
Box plots of significant among-group differences. **(A)** PHQ, **(B)** GAD, **(C)** QOL, **(D)** NEO2, **(E)** NEO3, and **(F)** NEO4. MDD, major depressive disorder; MDS, mild depressive symptoms; CON, control; PHQ, Patient Health Questionnaire-9; GAD, General Anxiety Disease-7; QOL, WHO quality of life; NEO2, NEO conscientiousness; NEO3, NEO extraversion; NEO4, NEO neuroticism.

One-way ANOVA was used to compare the MDD, MDS, and CON groups; the depression [*F*(2, 97) = 23.26, *P* < 0.001], anxiety [*F*(2, 97) = 15.36, *P* < 0.001], the QOL [*F*(2, 97) = 18.66, *P* < 0.001], conscientiousness [*F*(2, 97) = 3.17, *P* = 0.046], extraversion [*F*(2, 97) = 3.96, *P* = 0.022], and neuroticism [*F*(2, 97) = 13.59, *P* < 0.001] parameters differed significantly, as reported previously (25) (for the one-way ANOVA results only). Box plots of variables exhibiting significant group differences are shown in Figure 1.

The correlations between person-level variables are shown in Table 2. The PSQI revealed significant relationship with depression (*r* = 0.34, *P* < 0.001), anxiety (*r* = 0.33, *P* < 0.001), QOL (*r* = −0.33, *P* < 0.001), and neuroticism (*r* = 0.22, *P* = 0.025) scales. However, person-level time in bed and sleep efficiency indicators did not show a significant correlation with the aforementioned variables. Moreover, the association between PSQI, time in bed, and sleep efficiency was not significant.

**TABLE 2:** Pairwise relationships between person-level variables.

	1	2	3	4	5	6	7	8	9	10	11	12	13	14
(1) Sex	1													
(2) Age	–0.13	1												
(3) BED	–0.02	–0.03	1											
(4) EFFI	0.08	–0.05	0.05	1										
(5) PHQ	–0.02	0.02	0.18	0.09	1									
(6) GAD	-0.02	0.07	0.00	0.04	0.71***	1								
(7) QOL	0.18	–0.09	0.06	–0.14	–0.51***	–0.52***	1							
(8) NEO1	0.23*	0.12	0.09	0.09	0.02	–0.09	0.27**	1						
(9) NEO2	0.02	0.04	0.08	–0.12	–0.26**	–0.05	0.23*	0.03	1					
(10) NEO3	0.14	–0.04	0.07	-0.29**	–0.34***	–0.18	0.37***	0.11	0.17	1				
(11) NEO4	0.00	0.00	0.07	0.05	0.61***	0.65***	–0.55***	–0.14	–0.27**	–0.24*	1			
(12) NEO5	0.04	0.00	-0.14	–0.12	0.09	0.08	0.04	0.05	0.01	0.17	0.05	1		
(13) BIS	0.07	–0.06	0.10	–0.07	0.18	0.11	–0.26**	–0.19	–0.56***	0.13	0.27**	0.02	1	
(14) PSQI	0.12	–0.15	0.05	0.03	0.34***	0.33***	–0.33***	0.01	0.06	–0.05	0.22*	0.08	0.12	1

BED, time in bed; EFFI, sleep efficiency; PHQ, Patient Health Questionnaire-9; GAD, General Anxiety Disease-7; QOL, WHO quality of life; NEO1, NEO agreeableness; NEO2, NEO conscientiousness; NEO3, NEO extraversion; NEO4, NEO neuroticism; NEO5, NEO openness to experience; BIS, Barratt Impulsiveness Scale-11; PSQI, Pittsburgh Sleep Quality Index.

*P < 0.05; **P < 0.01; ***P < 0.001.

**FIGURE 2:**
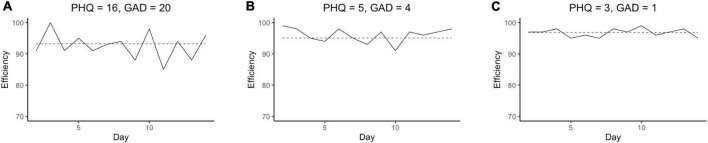
The trace plots for sleep efficiency of participant 23 in MDD group **(A)**, participant 31 in MDS group **(B)**, and participant 3 in CON group **(C)**. Solid line represents each participant’s daily sleep efficiency and dashed line indicates average sleep efficiency for each participant. PHQ, Patient Health Questionnaire-9; GAD, General Anxiety Disease-7; MDD, major depressive disorder; MDS, mild depressive symptoms; CON, control.

Figure 2 is the trace plots of daily sleep efficiencies of three participants. These participants manifest slightly different levels of mean sleep efficiency. However, they show substantial difference in variability in sleep efficiency. The participant in the Figure 2A, shows greater “ups and downs” of sleep efficiency (around the mean) compared to those in the Figure 2B, which in turn shows more fluctuation in sleep efficiency than those shown in the Figure 2C.

Table 3 summarizes the results of the DSEM analysis. Greater intra-individual variability in daily sleep efficiency was associated with higher levels of depression (*b* = 2.526, 95% CI = [0.221, 5.084]) and anxiety (*b* = 3.696, 95% CI = [1.313, 6.292]). However, the mean daily sleep efficiency did not show a significant relationship with depression (*b* = 0.292, 95% CI = [−0.387, 1.061]) and anxiety (*b* = 0.618, 95% CI = [−0.072, 1.412]). These results suggest that fluctuation in night-to-night sleep efficiency may be more important in terms of the development of depression and anxiety, rather than the average level of sleep efficiency. Longer bedtime, lower QOL, less extraversion, and more neuroticism were significantly associated with higher levels of depression (for bedtime, *b* = 0.024, 95% CI = [0.008, 0.04]; for QOL, *b* = −0.114, 95% CI = [−0.224, −0.003]; for extraversion, *b* = −0.14, 95% CI = [−0.27, −0.008]; for neuroticism, *b* = 0.223, 95% CI = [0.11, 0.341]). Lower QOL and greater neuroticism were associated with higher anxiety (for QOL, *b* = −0.112, 95% CI = [−0.221, −0.002]; for neuroticism *b* = 0.314, 95% CI = [0.203, 0.423]).

**TABLE 3:** The results of the dynamic structural equation modeling (DSEM) analysis.

		Posterior median	Posterior standard deviation	95% credible interval	Significance
**Outcome**	**Predictor**				
PHQ	MEAN EFFI	0.292	0.367	[–0.387, 1.061]	
	VAR EFFI	2.526	1.235	[0.221, 5.084]	*
	SEX	0.225	0.859	[–1.468, 1.892]	
	AGE	0.045	0.128	[–0.2, 0.303]	
	BED	0.024	0.008	[0.008, 0.04]	*
	QOL	–0.114	0.057	[–0.224, -0.003]	*
	NEO1	0.081	0.074	[–0.065, 0.225]	
	NEO2	–0.097	0.071	[–0.237, 0.045]	
	NEO3	–0.14	0.066	[–0.27, –0.008]	*
	NEO4	0.223	0.058	[0.11, 0.341]	*
	NEO5	0.074	0.059	[–0.042, 0.191]	
	BIS	–0.039	0.046	[–0.131, 0.05]	
	PSQI	0.259	0.154	[–0.04, 0.566]	
GAD	MEAN EFFI	0.618	0.376	[–0.072, 1.412]	
	VAR EFFI	3.696	1.263	[1.313, 6.292]	*
	SEX	0.188	0.831	[–1.439, 1.826]	
	AGE	0.142	0.125	[–0.101, 0.395]	
	BED	0.004	0.008	[–0.012, 0.02]	
	QOL	–0.112	0.056	[–0.221, –0.002]	*
	NEO1	–0.019	0.073	[–0.16, 0.124]	
	NEO2	0.083	0.07	[–0.057, 0.217]	
	NEO3	0.014	0.065	[–0.114, 0.14]	
	NEO4	0.314	0.056	[0.203, 0.423]	*
	NEO5	0.014	0.059	[–0.102, 0.13]	
	BIS	–0.04	0.046	[–0.128, 0.05]	
	PSQI	0.204	0.149	[–0.083, 0.496]	

PHQ, Patient Health Questionnaire-9; GAD, General Anxiety Disease-7; MEAN EFFI, individual mean of daily sleep efficiency; VAR EFFI, log-transformed individual variance of daily sleep efficiency; BED, time in bed; QOL, WHO quality of life; NEO1, NEO agreeableness; NEO2, NEO conscientiousness; NEO3, NEO extraversion; NEO4, NEO neuroticism; NEO5, NEO openness to experience; BIS, Barratt Impulsiveness Scale-11; PSQI, Pittsburgh Sleep Quality Index. Significance (*) indicates the 95% credible interval does not contain 0.

## Discussion

We explored whether variability in terms of daily sleep efficiency measured using wearable devices predicted depression and anxiety levels; we controlled for demographic and clinical characteristics. DSEM revealed that greater variability of daily sleep efficiency was associated with higher depression and anxiety levels than was average daily sleep efficiency, consistent with the findings of previous studies; night-to-night sleep fluctuations affect disease risk (9, 12, 13).

However, a study examined the relationship between intra-individual variability in daily subjective sleep quality and positive affect was found that people with higher fluctuations in daily sleep quality had more variability and higher mean positive affect than those who did not (49). These results are consistent with our study in terms of variability but are contrary to in terms of average. Therefore, in future studies, it is necessary to closely examine the associations between the objective and subjective aspects of the sleep quality and the level of depression and anxiety. Specifically, it is important to collect longitudinal sleep data in order to comprehensively consider the variability and mean values.

Wearable devices yield objective sleep measurements via real-time recording at minimal expense, and do not burden users (12); such devices facilitate the personalized and interactive healthcare (50). In this study, we used Fitbit Charge 2 as the wearable device and a previous study showed that sleep parameters measured with Fitbit devices were not statistically different from those measured with actigraphy (51); actigraphy has been used in chronobiology and sleep medicine for the past 20 years. However, some authors have questioned the validity of the raw data. In a previous experiment, sleep efficiency was high and varied only slightly (16); it was concluded that raw sleep efficiency data were not helpful. However, we found that raw sleep efficiency (daily variability) data predicted levels of depression and anxiety, although the sleep efficiencies of all groups were high. The daily (longitudinal) fluctuations in sleep efficiency revealed by wearable devices are valuable. Our research method can be easily utilized for sleep tracking to understand the temporal relationship between sleep efficiency routines and depression severity. Further, other sleep parameters such as sleep duration and times to fall asleep may be used to speculate associations with sleep routines and related health outcomes (52).

Furthermore, ANOVA revealed significant group differences in depression, anxiety, the subjective QOL, and personality traits; neither objective nor subjective sleep quality differed among the groups. Notably, the PSQI score exceeded 7 regardless of the severity of depression, indicating poor sleep quality. One study found that most American college students have poor sleep quality, explained principally by perceived stress (53); similar results were obtained in work on Portuguese college students in whom relationships were apparent among perceived stress, sleep difficulties, affect, and rumination (54). Thus, the high PSQI scores of our participants may reflect high stress rather than depression. Interestingly, PSQI was significantly correlated with PHQ, which looks incompatible with the non-significant group difference found in ANOVA. A further study is needed to investigate this issue, but we conjecture that classifying the MDD/MDS/CON groups yielded loss of information about individual difference in the severity of depression symptoms within groups, and it may have reduced the statistical power to detect the association between PSQI and depression (55).

Additionally, the objective sleep efficiency exceeded 90% for most participants, and it means sleep quality was good (27). The difference between subjective and objective sleep quality was apparent in correlation analysis. The PSQI score significantly correlated with depression, anxiety, the subjective QOL, and neuroticism but not sleep efficiency. Besides, we found a low correlation between PSQI and sleep efficiency. A non-significant association between subjective and objective sleep quality was found in previous studies (56, 57) in which psychological and physical discrepancies were also evident. Subjective sleep quality seems to be affected by psychological health (56); our DSEM finding that PSQI did not predict the PHQ or GAD score could be viewed as an extension of such results.

The significance of our findings is that depression and anxiety levels can be predicted by variability of daily sleep efficiency measured with wearable devices. This implies that the variability in sleep efficiency is an effective measure of individual’s mental health. Therefore, monitoring mental health using wearable devices in a cost-effective and interactive manner with focusing on variability in sleep quality across multiple days could help detect depression and anxiety in young adults.

## Limitations

Our work had certain limitations. First, we explored whether variability in daily sleep efficiency and clinical characteristics predicted depression or anxiety but the depression, anxiety, and clinical scores were measured prior to assessment of daily sleep efficiency. Thus, we cannot infer a true temporal precedence between irregular daily sleep quality and depressive symptoms. A study on the temporal relationship between day-to-day variability in sleep efficiency and depression severity is required. Second, we collected only objective sleep data on a daily basis. A future study should collect both day-to-day clinical characteristics (such as mood state and subjective sleep quality) and objective sleep quality information. Third, all of our participants were undergraduate or graduate students, thus not representative sample of all young adults. A future study on young adults engaged in various occupations is necessary. Finally, some demographic factors were not collected. Residential status, body mass index, and physical condition may affect sleep quality.

## Conclusion

In conclusion, young adults exhibiting greater daily variability in objective sleep efficiency may be at high risk for depression; longitudinal monitoring is required. Interventions should consider the magnitudes of day-to-day fluctuations in objective sleep quality and the self-reported psychological profiles.

## Data availability statement

The original contributions presented in this study are included in the article/supplementary material, further inquiries can be directed to the corresponding authors.

## Ethics statement

The studies involving human participants were reviewed and approved by Institutional Review Board of the Seoul National University College of Medicine and Hospital (Seoul, South Korea; approval no. 1608–079–785). The patients/participants provided their written informed consent to participate in this study.

## Author contributions

J-AL: methodology, data analysis, and writing—original draft. J-YY and S-HC: recruit subjects, acquisition of data, and revision. SP: acquisition of data. HS: methodology, revision, editing, and supervision. JJ: project administration, conceptualization, funding acquisition, recruited subjects, and revision. All authors contributed to the article and approved the submitted version.

## Abbreviations

PSG: polysomnography
MDD: major depressive disorder
MDS: mild depressive symptoms
CON: control
BED: time in bed
EFFI: sleep efficiency
MEAN EFFI: individual mean of daily sleep efficiency
VAR EFFI: log-transformed individual variance of daily sleep efficiency
PHQ: Patient Health Questionnaire-9
GAD: General Anxiety Disease-7
QOL: WHO quality of life
NEO1: NEO agreeableness
NEO2: NEO conscientiousness
NEO3: NEO extraversion
NEO4: NEO neuroticism
NEO5: NEO openness to experience
BIS: Barratt Impulsiveness Scale-11
PSQI: Pittsburgh Sleep Quality Index
iSD: intra-individual standard deviation
DSEM: dynamic structural equation modeling.
